# Pretreatment Serum Concentrations of 25-Hydroxyvitamin D and Breast Cancer Prognostic Characteristics: A Case-Control and a Case-Series Study

**DOI:** 10.1371/journal.pone.0017251

**Published:** 2011-02-28

**Authors:** Song Yao, Lara E. Sucheston, Amy E. Millen, Candace S. Johnson, Donald L. Trump, Mary K. Nesline, Warren Davis, Chi-Chen Hong, Susan E. McCann, Helena Hwang, Swati Kulkarni, Stephen B. Edge, Tracey L. O'Connor, Christine B. Ambrosone

**Affiliations:** 1 Department of Cancer Prevention and Control, Roswell Park Cancer Institute, Buffalo, New York, United States of America; 2 Department of Social and Preventive Medicine, University at Buffalo, Buffalo, New York, United States of America; 3 Department of Pharmacology and Therapeutics, Roswell Park Cancer Institute, Buffalo, New York, United States of America; 4 Department of Medicine, Roswell Park Cancer Institute, Buffalo, New York, United States of America; 5 Department of Pathology, Roswell Park Cancer Institute, Buffalo, New York, United States of America; 6 Department of Surgical Oncology, Roswell Park Cancer Institute, Buffalo, New York, United States of America; Brigham & Women's Hospital, and Harvard Medical School, United States of America

## Abstract

**Background:**

Results from epidemiologic studies on the relationship between vitamin D and breast cancer risk are inconclusive. It is possible that vitamin D may be effective in reducing risk only of specific subtypes due to disease heterogeneity.

**Methods and Findings:**

In case-control and case-series analyses, we examined serum concentrations of 25-hydroxyvitamin D (25OHD) in relation to breast cancer prognostic characteristics, including histologic grade, estrogen receptor (ER), and molecular subtypes defined by ER, progesterone receptor (PR) and HER2, among 579 women with incident breast cancer and 574 controls matched on age and time of blood draw enrolled in the Roswell Park Cancer Institute from 2003 to 2008. We found that breast cancer cases had significantly lower 25OHD concentrations than controls (adjusted mean, 22.8 versus 26.2 ng/mL, p<0.001). Among premenopausal women, 25OHD concentrations were lower in those with high- versus low-grade tumors, and ER negative versus ER positive tumors (p≤0.03). Levels were lowest among women with triple-negative cancer (17.5 ng/mL), significantly different from those with luminal A cancer (24.5 ng/mL, p = 0.002). In case-control analyses, premenopausal women with 25OHD concentrations above the median had significantly lower odds of having triple-negative cancer (OR = 0.21, 95% CI = 0.08–0.53) than those with levels below the median; and every 10 ng/mL increase in serum 25OHD concentrations was associated with a 64% lower odds of having triple-negative cancer (OR = 0.36, 95% CI = 0.22–0.56). The differential associations by tumor subtypes among premenopausal women were confirmed in case-series analyses.

**Conclusion:**

In our analyses, higher serum levels of 25OHD were associated with reduced risk of breast cancer, with associations strongest for high grade, ER negative or triple negative cancers in premenopausal women. With further confirmation in large prospective studies, these findings could warrant vitamin D supplementation for reducing breast cancer risk, particularly those with poor prognostic characteristics among premenopausal women.

## Introduction

Vitamin D is a secosteroid hormone critical to bone health and other biological pathways [1]. Circulating 25-hydroxyvitamin D (25OHD), the widely-used biomarker for endogenous levels of vitamin D, as well as proxies of vitamin D exposure, such as sun exposure and dietary and supplementary intake, have been evaluated in relation to risk of various malignancies [2]. However, consistent associations have only been demonstrated for colorectal cancer [3], [4]. Despite numerous experimental studies repeatedly showing anti-neoplastic activities of vitamin D on breast cancer [5], [6], findings from epidemiologic studies and randomized trials are not definitive [7], [8], [9].

It is possible that tumor heterogeneity in breast cancer may mask associations. Clinical markers including estrogen receptor (ER), progesterone receptor (PR) and tumor grade have long been used to classify breast cancer subtypes associated with differential prognosis and response to cancer therapy. These crude subtypes were refined by recent gene expression microarray studies, which clustered breast tumors into five major molecular subtypes [10], [11]. A validated panel of immunohistochemical (IHC) markers have been developed to approximate the classification of these subtypes, including luminal A (ER+ and/or PR+ and HER2-), luminal B (ER+ and/or PR+ and either HER2+ or Ki-67+), non-luminal HER2+ (ER-, PR-, and HER2+), basal-like (ER-, PR-, HER2-, CK5/6+ and/or HER1+), and unclassified (ER-, PR-, HER2-, CK5/6-, and HER1-) [12], [13], [14]. Several studies have shown that reproductive risk factors differ for the particular molecular subtypes [15], [16], [17]; and it is likely that relationships between vitamin D and breast cancer risk may also vary according to subtypes. Interestingly, in the Physicians' Health Study, blood levels of 1,25-dihydroxyvitamin D were strongly associated with the risk of aggressive, but not total prostate cancer [18]. Similar differential associations may also exist for breast cancer.

The definition of breast tumors that are ‘triple negative’, i.e., lack of expression of ER, PR and HER2, largely overlap with that of basal-like tumors and is sometimes used as a proxy for the latter. Basal-like or triple negative tumors pose a major challenge for breast cancer treatment, because it does not repond to hormonal therapy targeting ER or trastuzumab targeting HER2. In a recent case-series study, women with triple negative breast cancer had the lowest serum 25OHD concentrations compared to those with other molecular cancer subtypes [19]. However, only 15 patients with triple negative cancer were included in that analysis. In a case-controls study of 579 women with primary incident breast cancer and 574 controls matched on age and time of blood collection, we examined serum concentrations of 25OHD at diagnosis or enrollment, with a particular focus on associations with breast cancer prognostic characteristics, specifically, tumor histologic grade, ER status, and molecular subtypes characterized by ER, PR and HER2.

## Methods

### Study population

Data and specimens from women with breast cancer and healthy controls were obtained from the Data Bank and Biorepository (DBBR) at Roswell Park Cancer Institute (RPCI). The DBBR, as previously described [20], is a comprehensive data and sample bank containing pretreatment biospecimens that are rigorously collected and processed, with comprehensive clinical and epidemiologic data. Briefly, patients newly diagnosed with cancer at RPCI are invited to participate during their initial visit with the surgical oncologist. After consent, blood samples are collected (prior to any treatment, including surgery, for breast cancer) in phlebotomy when specimens for clinical measures are drawn, transported to the laboratory through a pneumatic tube system, and processed within one hour of blood draw. Specimens are maintained in liquid nitrogen until analysis. The average time interval between the time of diagnosis and the time of blood draw for the women in our study was 27 days.

Inclusion criteria for breast cancer cases in the study were: self-identified as non-Hispanic white, histologically confirmed primary, incident, female breast cancer, and no prior cancer history except non-melanoma skin cancer. Healthy controls were identified from family members and friends of the patients and other visitors to RPCI or from volunteers recruited from community events, and blood was drawn and processed at RPCI in the same manner as the cases. For this study, controls were matched to cases on five year age category and month of blood collection. Those who were family members or friends of the breast cancer cases were not included in the study. Self-administered questionnaires were used to collect data on demographics, reproduction, medical history, family histories of cancer, and lifestyle factors including physical activity. Self-reported physical activity compared to same age peers was used as an estimate for sun exposure. In addition, questionnaire data on activities including walking, running, cycling, and golfing were included as alternative estimates for sun exposure. A food frequency questionnaire was administered, and questions on supplement use were included. Ninety-two percent (92%) of the women in this study had questionnaires returned. Postmenopausal status in the study was defined as women who experienced 12 consecutive months of amenorrhea, or women who underwent bilateral salpingo-oophorectomy. This study was approved by the Institutional Review Board at RPCI.

### Clinical data and breast cancer prognostic characteristics

Patients' clinical data, including tumor stage, histologic grade and ER, PR and HER2 status, were obtained from a clinical database maintained by the RPCI breast program, and supplemented with data from abstracted medical records and the RPCI Tumor Registry. Because IHC of CK 5/6 or EGFR was not routinely performed in pathology, we instead defined four molecular subtypes in our study based on ER, PR and HER2 as follows: luminal A (ER+ and/or PR+, HER2-), luminal B (ER+ and/or PR+, HER2+), non-luminal HER2+ (ER-, PR- and HER2+), and triple negative (ER-, PR- and HER2-). As such, we were not able to distinguish the basal-like and unclassified subtypes, both of which were included in the triple negative group in our study. However, it has been shown that the prognostic significance of the triple negative subtype is similar to that of the basal-like subtype [21]. ER, PR and HER2 status were measured by IHC in pathology, and amplification of *HER2* gene was tested by fluorescence *in situ* hybridization (FISH) when IHC scored 2+. Histologic grade, ER status and molecular subtypes defined by ER, PR and HER2 were used as three independent prognostic characteristics for breast cancer. In addition, tumor stage was also included for analysis.

### Serum 25-hydroxyvitamin D assay

Considering the potential variability of 25OHD assays, we first tested assay performance on pilot samples in two different laboratories both running the immunochemiluminometric assay on the DiaSorin Liasion automated instrument. At one laboratory, the coefficient of variation (CV) was 19%, which was considered inappropriate for the purpose of this study. At another laboratory (Heartland Assay, Ames, IA), the CV was 6.5%, and this laboratory was chosen. For the entire batch of samples analyzed for cases and controls, the CV was 8.8%.

### Statistical analysis

For univariate analysis, we first compared serum 25OHD concentrations in the healthy controls by a number of selected factors that might affect vitamin D levels, using non-parametric tests. To compare serum 25OHD concentrations by case-control status or by tumor prognostic characteristics, we used a generalized linear model controlling for age, body mass index (BMI) and season of blood collection, which had independent effects on serum 25OHD concentrations (p<0.05). Least square means and standard errors of 25OHD concentrations were derived separately for each of the tumor characteristics. Physical activity was not associated with serum 25OHD levels after control for BMI, and adding it to the models had little impact on the results. Thus, results without additional adjustment for physical activity are presented.

To examine serum 25OHD levels in relation to breast cancer prognostic characteristics, we performed two types of analyses, including case-control analysis, where healthy controls were used as a referent group, and case-series analysis, where women with better prognostic characteristics (grade I/II, ER+, or luminal A subtype) were used as a referent group and women with carcinoma *in situ* (CIS) were excluded. Logistic regression was used to estimate odds ratios (OR) and 95% confidence intervals (CI) associated with 25OHD levels. When outcomes had more than two levels, multinomial logistic regression models were fitted. Considering large seasonal variations of 25OHD concentrations due to change of solar ultra-violet B intensity in the Northeastern United States through a year, we computed season-standardized 25OHD concentrations by locally weighted multinomial regression to determine the cut-off points of vitamin D levels for logistic regression, following the approach described by Ahn and colleagues [22].

For case-control analysis, season-standardized vitamin D levels were defined as follows: deficient (<20.0 ng/mL), insufficient (20.0–29.9 ng/mL), and sufficient (≥30.0 ng/mL). For case-series analysis of prognostic characteristics, because the number of cases was limited in some categories, we dichotomized season-standardized 25OHD concentrations based on the medians in healthy controls. In addition, we also treated season-standardized 25OHD concentrations as a continuous variable in the regression models and computed the ORs and 95% CIs associated with an incremental increase of 10 ng/mL of 25OHD. Because etiologic pathways of breast cancer may differ between premenopausal and postmenopausal women, we first performed analyses for all women, and then stratified the analyses by menopausal status. All analyses were performed using SAS 9.2 with two-sided significance level of 0.05 (SAS Institute, Cary, NC).

## Results

Table 1 shows serum 25OHD concentrations according to selected demographic and lifestyle characteristics of the control population. Younger women tended to have higher 25OHD levels than older women, although the differences were not statistically significant. There were apparent seasonal variations of serum 25OHD concentrations, with a peak during summer season. Circulating 25OHD concentrations were inversely associated with BMI, and positively associated with physical activity. Women who had higher dietary vitamin D intake or took vitamin D supplements had higher circulating concentrations.

**Table 1:** Serum 25-hydroxyvitamin D concentrations by demographic and lifestyle characteristics among healthy controls.

Characteristics	N (%)^1^	Serum 25OHD, median (IQR), ng/ml	P-value^2^
Age, year			0.56
<50	202 (35.2)	28.3 (19.8–36.3)	
50–59	169 (29.4)	27.0 (19.2–33.4)	
60–69	127 (22.1)	26.8 (19.4–32.3)	
≥70	76 (13.2)	26.7 (19.8–33.5)	
Season of blood collection			<0.001
Spring (Mar–May)	99 (17.2)	25.7 (15.9–33.2)	
Summer (Jun–Aug)	175 (30.5)	30.5 (22.9–36.9)	
Fall (Sep–Nov)	135 (23.5)	25.2 (19.5–32.8)	
Winter (Dec–Feb)	165 (28.7)	24.7 (16.5–31.8)	
BMI, kg/m^2^			<0.001
<25.0	184 (33.0)	30.7 (24.4–38.8)	
25.0–29.9	198 (35.5)	27.5 (20.7–33.2)	
≥30.0	175 (31.4)	21.6 (15.4–28.2)	
Physical activity			<0.001
More active	264 (46.2)	29.3 (22.2–36.5)	
Normal	198 (34.5)	24.3 (18.8–31.9)	
Less active	110 (19.2)	25.1 (16.1–33.2)	
Dietary vitamin D			0.003
Q1 (<42 IU/day)	146 (25.4)	24.0 (17.8–32.2)	
Q2 (42–147 IU/day)	134 (23.3)	27.3 (20.6–36.8)	
Q3 (148–329 IU/day)	142 (24.7)	27.6 (20.6–32.8)	
Q4 (≥330 IU/day)	152 (26.5)	27.3 (21.0–37.6)	
Supplementary vitamin D			<0.001
Yes	259 (45.1)	28.1 (22.2–35.0)	
No	315 (54.9)	24.9 (16.6–33.0)	

1 For some characteristics, the numbers did not add up to the totals due to missing data.

2 P-values were derived from Wilcoxon rank test for variables with two levels and Kruskal-Wallis test for variables with more than two levels. Abbreviation: IQR, interquartile range.

The median of serum 25OHD concentrations in breast cancer cases and controls were 22.8 ng/mL and 26.2 ng/mL, respectively. After control for seasonal variations, a majority of the controls were either vitamin D deficient (25.8%) or insufficient (35.7%), and only 38.5% of them had a sufficient level of 30 ng/mL or higher. The proportion of vitamin D deficiency was even higher in cases (38.5%), and only a small proportion of them were considered vitamin D sufficient (21.4%) (p<0.001). As shown in Table 2, compared to women who were vitamin D deficient, those with sufficient levels had a 63% reduction in odds of breast cancer (OR = 0.37, 95% CI = 0.27–0.51). Every 10 ng/mL incremental increase of 25OHD concentrations was associated with an estimated reduction of breast cancer odds by one third (OR = 0.67, 95% CI = 0.59–0.75), which was significant in both premenopausal and postmenopausal women.

**Table 2:** Odds ratios and 95% confidence intervals for breast cancer by serum 25-hydroxyvitamin D levels.

Serum 25OHD levels	All			Premenopausal			Postmenopausal		
	case n (%)	control n (%)	OR (95% CI)	case n (%)	control n (%)	OR (95% CI)	case n (%)	control n (%)	OR (95% CI)
Deficient	220 (38)	156 (27)	1.00	82 (33)	74 (30)	1.00	138 (41)	82 (25)	1.00
Insufficient	241 (42)	203 (35)	0.81 (0.61–1.08)	110 (45)	83 (34)	1.13 (0.72–1.77)	131 (39)	120 (36)	0.64 (0.44–0.94)
Sufficient	118 (20)	215 (37)	0.37 (0.27–0.51)	53 (22)	88 (36)	0.57 (0.34–0.93)	65 (19)	127 (39)	0.29 (0.19–0.45)
P-value for trend			<0.001			0.03			<0.001
Continuous per 10 ng/mL increment	579	574	0.67 (0.59–0.75)	245	245	0.76 (0.63–0.91)	334	329	0.61 (0.52–0.72)

1 Serum 25-hydroxyvitamin D (25OHD) concentrations were adjusted by the week of blood collection time in a year by locally weighted multinomial regression. The three levels were defined as follows: deficient, <20.0 ng/mL; insufficient, 20.0–29.9 ng/mL; sufficient, ≥30.0 ng/mL.

2 Odds ratios (OR) ad 95% confidence intervals (CI) were adjusted for age and BMI. Further adjustment for physical activity did not significantly change the results (data not shown).

When pre- and postmenopausal women with invasive breast cancer were considered together, there were no significant differences in serum 25OHD concentrations by histologic grade or ER status (data not shown). However, women with triple negative breast cancer had the lowest vitamin D concentrations among the 4 molecular subtypes after control for age, BMI and season of blood collection (least square mean ± standard error: 23.0±0.5, 21.3±1.3, 21.6±1.6 and 19.9±1.1 ng/mL for luminal A, luminal B, non-luminal HER2+ and triple negative subtypes, respectively, p = 0.046). In addition, there was an inverse relationship between serum 25OHD concentrations and tumor stage (26.5±1.0, 23.2±0.5, 21.3±0.7 and 21.9±2.0 ng/mL for stage 0 [CIS], stage I, stage II/IIIA, and stage IIIB/IIIC/IV, respectively, p<0.001).

When stratifying by menopausal status, serum 25OHD levels did not differ by tumor characteristics among postmenopausal women, but there were notable differences among premenopausal women (Table 3). Those diagnosed with invasive breast cancer, especially late stage cancer, had significantly lower 25OHD concentrations than those with CIS (p<0.001). Among premenopausal women with invasive breast cancer, those who had high grade or ER negative cancer had lower serum 25OHD concentrations than those with high grade or ER positive cancer (p≤0.03). Moreover, premenopausal women diagnosed with triple negative cancer tumors had the lowest concentrations compared to those with the other three molecular subtypes (p = 0.002).

**Table 3:** Serum 25-hydroxyvitamin D concentrations by prognostic characteristics in premenopausal and postmenopausal women diagnosed with breast cancer.

Tumor characteristics	All (n = 579)	Premenopausal women (n = 245)			Postmenopausal women (n = 334)		
	N (%)^1^	N (%)^1^	mean ± se^2^, ng/mL	P-value	N (%)^1^	mean ± se^2^, ng/mL	P-value
Tumor stage				<0.001			0.23
In situ	86 (15)	42 (17)	28.9±1.4		44 (13)	24.8±1.4	
I	292 (51)	95 (39)	24.8±0.9		197 (59)	22.3±0.7	
II/IIIA	179 (31)	96 (39)	21.3±1.0		83 (25)	21.4±1.0	
IIIB/IIIC/IV	20 (3)	11 (5)	20.0±2.7		9 (3)	24.4±3.0	
Histologic grade				0.005			0.81
I/II	166 (35)	56 (29)	26.0±1.3		110 (40)	21.9±0.8	
III	305 (65)	137 (71)	21.6±0.8		168 (60)	22.1±0.7	
ER status				0.03			0.76
Positive	372 (76)	147 (73)	23.7±0.8		225 (79)	22.1±0.6	
Negative	115 (24)	55 (27)	20.2±1.3		60 (21)	21.7±1.2	
Molecular subtype				0.002			0.92
Luminal A	330 (69)	129 (64)	24.5±0.8		201 (71)	22.2±0.6	
Luminal B	49 (10)	23 (11)	21.2±1.9		26 (9)	21.1±1.7	
Non-luminal HER2+	32 (6)	15 (7)	21.7±2.5		17 (6)	21.2±2.2	
Triple negative	74 (15)	34 (17)	17.5±1.6		40 (14)	21.8±1.4	

1 Two patients with tumor stage not evaluable (TX) were excluded from analysis of stage. For the analysis of histologic grade, ER status and molecular subtype, women with carcinoma *in situ* (n = 86) were excluded. The numbers do not add up to the total due to missing data: histologic grade (missing n = 22 or 4%), ER status (missing n = 6 or 1%), and molecular subtype (missing n = 8 or 2%).

2 Least square mean and standard error (se) were adjusted for age, season of blood collection, and body mass index in linear regression models. Additional adjustment for physical activity did not significantly change the results (data not shown).

**Figure 1:**
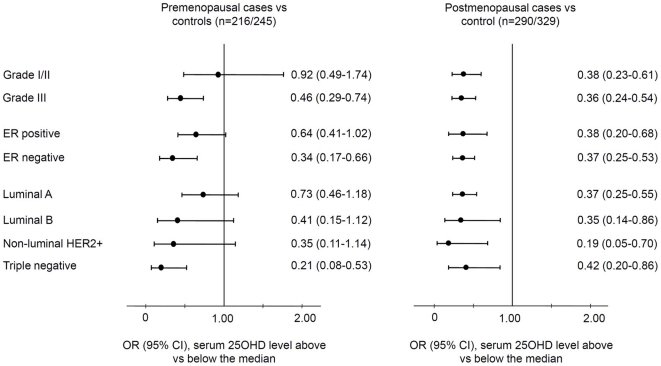
Case-control analysis of breast cancer risk by high and low vitamin D levels. Season-standardized serum 25-hydroxyvitamin D (25OHD) concentrations were stratified into above and below the median in healthy controls by menopausal status. Odds ratios (OR) and 95% confidence intervals (CI) were derived from multinomial logistic regression with adjustment for age at diagnosis and BMI, and presented in groups of tumor characteristics, where healthy controls were used as a referent group. Further adjustment for physical activity did not significantly change the results (data not shown). The lengths of horizontal lines are indicative of confidence intervals and the dots are indicative of odds ratios, with the corresponding odds ratios and 95% confidence interval given in numbers on the right of the Y-axis.

In case-control analyses, ORs and 95% CIs of breast cancer by menopausal status and tumor prognostic characteristics are plotted in Figure 1. Among premenopausal women, those with 25OHD concentrations above the median had significantly reduced odds of grade III cancer (OR = 0.46, 95% CI = 0.29–0.74), ER negative cancer (OR = 0.34, 95% CI = 0.17–0.66), and triple negative cancer (OR = 0.21, 95% CI = 0.08–0.53). Using continuous vitamin D data, an incremental increase of 10 ng/mL 25OHD concentrations was associated with about two thirds reduction of odds of triple negative breast cancer (OR = 0.36, 95% CI = 0.22–0.56) (Figure S1). Among postmenopausal women, higher serum vitamin D levels were associated with reduced odds of breast cancer regardless of tumor characteristics.

**Figure 2:**
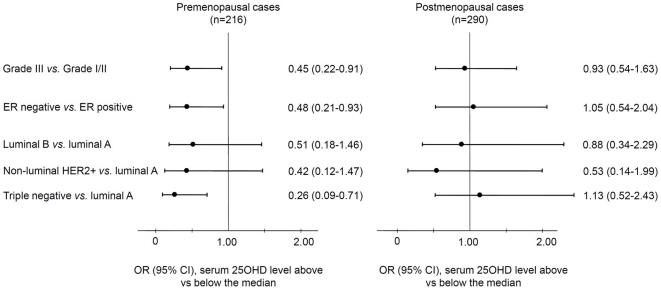
Case-series analysis of breast cancer risk by high and low vitamin D levels. Season-standardized serum 25-hydroxyvitamin D (25OHD) concentrations were stratified into above and below the median in healthy controls by menopausal status. Odds ratios (OR) and 95% confidence intervals (CI) were derived from multinomial logistic regression with adjustment for age at diagnosis and BMI, and presented in groups of tumor characteristics, where women with better prognostic characteristics (grade I/II, ER+, or luminal A subtype) were used as a referent group and women with carcinoma *in situ* (CIS) were excluded. Further adjustment for physical activity did not significantly change the results (data not shown). The lengths of horizontal lines are indicative of confidence intervals and the dots are indicative of odds ratios, with the corresponding odds ratios and 95% confidence interval given in numbers on the right of the Y-axis.

In case-series analyses, high levels of serum 25OHD were less likely to be associated with premenopausal breast cancer with poor prognostic characteristics than low levels (grade III versus I/II, OR = 0.45, 95% CI = 0.22–0.91; ER negative versus positive, OR = 0.48, 95% CI = 0.21–0.93; triple negative versus luminal A subtype, OR = 0.26, 95% CI = 0.09–0.71) (Figure 2). Similar results were also found with a 10 ng/mL incremental increase of serum 25OHD concentrations (Figure S2). In contrast, there were no associations of 25OHD levels with cancer prognostic characteristics in parallel analyses among postmenopausal women (Figures 2 and S2).

## Discussion

We found that higher serum 25OHD concentrations were associated with significantly reduced odds of both premenopausal and postmenopausal breast cancer. Among premenopausal women only, 25OHD concentrations were significantly lower in women with tumors with poor prognostic characteristics (high grade, ER negative, and triple negative) than among those with cancers with better prognostic features. The findings support the hypothesis that vitamin D may reduce risk of the development of a subset of tumors with more aggressive characteristics and poorer prognosis.

Existing evidence supports a link between vitamin D and prognostic characteristics of breast cancer. In clinical studies, serum 25OHD concentrations have been inversely associated with breast cancer stage [23], [24] and histologic grade [25]. In a multiethnic cohort of breast cancer survivors, women with ER negative breast cancer had significantly lower serum 25OHD than those with ER positive tumors [24], and in a case-control study with both pre- and postmenopausal women, reduced risk of breast cancer with higher 25OHD levels was found only among women with ER-/PR- tumors [26]. In a German case-control study of premenopausal women, plasma 25OHD and dietary vitamin D intake were more strongly related to ER- or PR- breast cancer than hormone receptor (HR) positive cancers [27], [28]. Similar to our findings, among postmenopausal women from the same study, the inverse relationships between 25OHD levels and breast cancer risk did not differ by HR status [29]. However, there are also studies with observed associations only for HR positive cancers [30], [31] or with null findings [32], [33].

The inconsistency in results across studies could be explained by heterogeneity in study populations, in classification of HR status, and/or in assessment of vitamin D status (dietary intake versus circulating 25OHD). Our patient population represents a more contemporary patient cohort (2003–2008) with data available on ER, PR and HER2, allowing us to refine tumor subtype classification and to distinguish the triple negative subtype. However, we were not able to classify the basal-like subtype due to lack of data on basal markers CK5/6 or EGFR. Although basal-like and triple negative phenotypes largely overlap and share a poor prognosis, the former definition represents a more refined group by excluding the unclassified subtype, which may behave differently in prognosis from the basal-like subtype. Our findings warrant validation in large prospective studies where complete data on molecular subtypes are available.

Although the exact biological mechanisms are not clear, data from animal experiments are concordant with our findings. *Vdr* knockout mice gavaged with the carcinogen dimethylbenzanthracene (DMBA) were more likely to develop ER-/PR- mammary tumors than wild type littermates [34]. Moreover, *VDR* expression were remarkably lower in ER- than in ER+ breast tumors [35], and the elevation of VDR nuclear corepressor NCoR1 level was particularly associated with ER negativity [36]. There are two possible explanations for these findings. First, vitamin D may prevent the occurrence of ER negative breast cancer by interfering with estrogen signaling pathway, as treatment with 1,25(OH)_2_D down-regulated the abundance of ER and suppressed estrogen activity in breast cancer cells [37], and vitamin D supplementation significantly reduced blood levels of progesterone and estradiol in women [38]. Second, vitamin D may prevent aggressive breast cancers by modulating the extracellular microenvironment, as vitamin D has been shown to alter the expression of a variety genes involved in extracelluar matrix remodeling [39], [40] and to modulate breast cancer phenotypes [41].

A limitation of our study is that only a single measurement of vitamin D at diagnosis was used, which may not necessarily represent vitamin D levels at the time of cancer initiation or progression. However, in a recent study, the correlation coefficient for measurement of 25OHD concentrations in serum samples collected in 1994 and 2008 ranged from 0.42 to 0.52, and was 0.80 when measured 12 months apart [41], suggesting reasonable stability of endogenous vitamin D status. Because blood samples in our study were collected shortly after diagnosis, prior to surgery or any adjuvant therapy, there would be little influence on vitamin D levels from life style changes after cancer diagnosis or from treatment.

In conclusion, our study provides compelling evidence that endogenous vitamin D levels may be associated with the etiology of breast cancer, particularly the triple negative subtype leading to poor prognosis among premenopausal women. Because the risk of triple negative breast cancer peaks before menopause, and because vitamin D deficiency can be easily corrected by increasing sun exposure and/or supplement intake, if our findings are confirmed in large prospective studies for temporal causality, vitamin D may be used as a potential cancer preventive agent against triple negative cancers among young women.

## Supporting Information

Figure S1 **Case-control analysis of breast cancer risk by 10 ng/ml increase of vitamin D levels.** Season-standardized serum 25-hydroxyvitamin D (25OHD) concentrations were entered into the regression models as a continuous variable. Odds ratios (OR) and 95% confidence intervals (CI) of an incremental increase of 10 ng/mL 25OHD were derived from multinomial logistic regression with adjustment for age at diagnosis and BMI, and presented in groups of tumor characteristics, where healthy controls were used as a referent group. Further adjustment for physical activity did not significantly change the results (data not shown). The lengths of horizontal lines are indicative of confidence intervals and the dots are indicative of odds ratios, with the corresponding odds ratios and 95% confidence interval given in numbers on the right of the Y-axis.
(TIF)

Figure S2 **Case-only analysis of breast cancer risk by 10 ng/ml increase of vitamin D levels.** Season-standardized serum 25-hydroxyvitamin D (25OHD) concentrations were entered into the regression models as a continuous variable. Odds ratios (OR) and 95% confidence intervals (CI) of an incremental increase of 10 ng/mL 25OHD were derived from multinomial logistic regression with adjustment for age at diagnosis and BMI, and presented in groups of tumor characteristics, where women with better prognostic characteristics (grade I/II, ER+, or luminal A subtype) were used as a referent group and women with carcinoma *in situ* (CIS) were excluded. Further adjustment for physical activity did not significantly change the results (data not shown). The lengths of horizontal lines are indicative of confidence intervals and the dots are indicative of odds ratios, with the corresponding odds ratios and 95% confidence interval given in numbers on the right of the Y-axis.
(TIF)
