# Supplementary figures and images for: Pretreatment Serum Concentrations of 25-Hydroxyvitamin D and Breast Cancer Prognostic Characteristics: A Case-Control and a Case-Series Study
Source: PLoS One. 2011 Feb 28;6(2):e17251. doi: 10.1371/journal.pone.0017251 (PMC3046139; doi:10.1371/journal.pone.0017251)

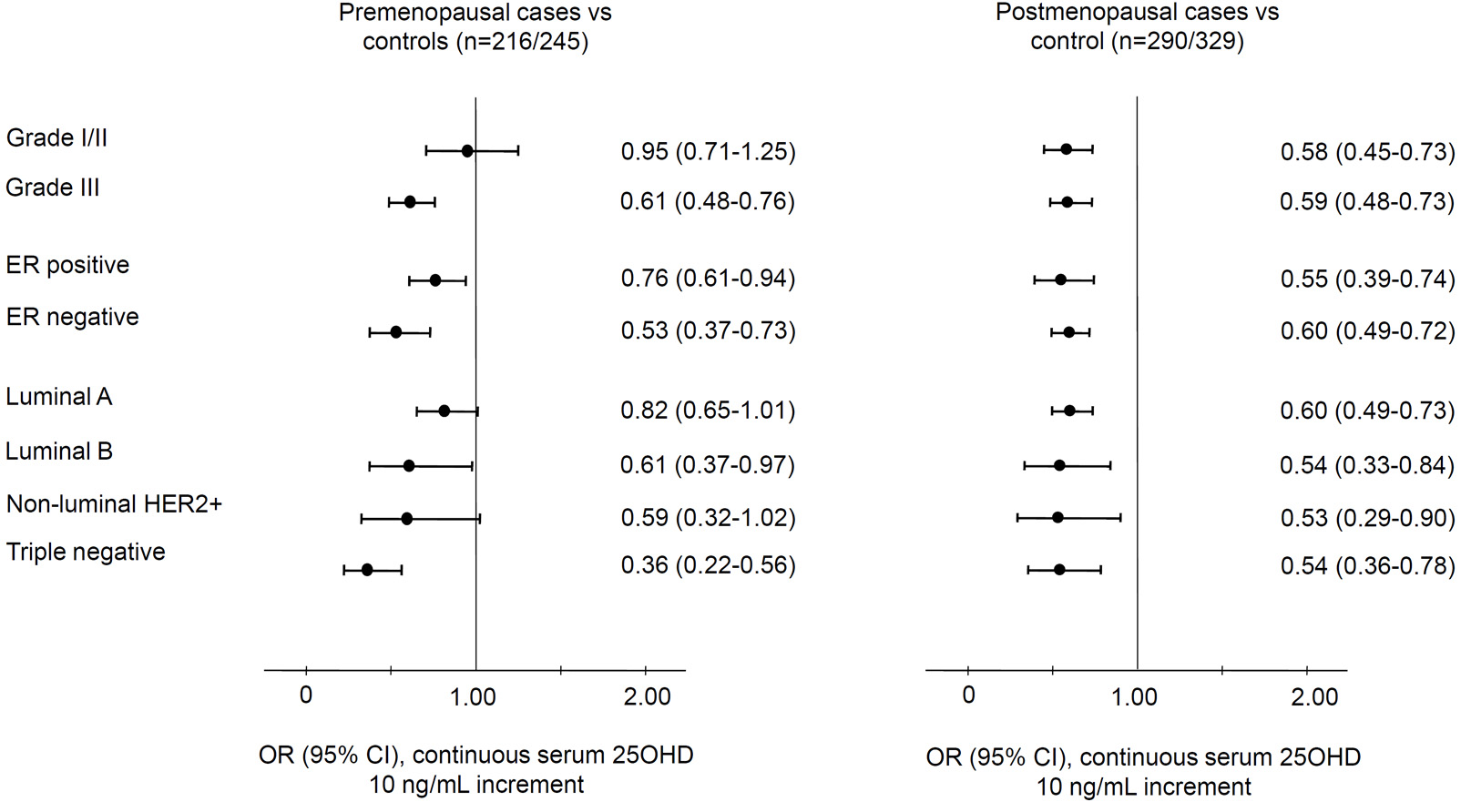

Supplement: Figure S1 — Case-control analysis of breast cancer risk by 10 ng/ml increase of vitamin D levels. Season-standardized serum 25-hydroxyvitamin D (25OHD) concentrations were entered into the regression models as a continuous variable. Odds ratios (OR) and 95% confidence intervals (CI) of an incremental increase of 10 ng/mL 25OHD were derived from multinomial logistic regression with adjustment for age at diagnosis and BMI, and presented in groups of tumor characteristics, where healthy controls were used as a referent group. Further adjustment for physical activity did not significantly change the results (data not shown). The lengths of horizontal lines are indicative of confidence intervals and the dots are indicative of odds ratios, with the corresponding odds ratios and 95% confidence interval given in numbers on the right of the Y-axis. (TIF) [file pone.0017251.s001.tif]

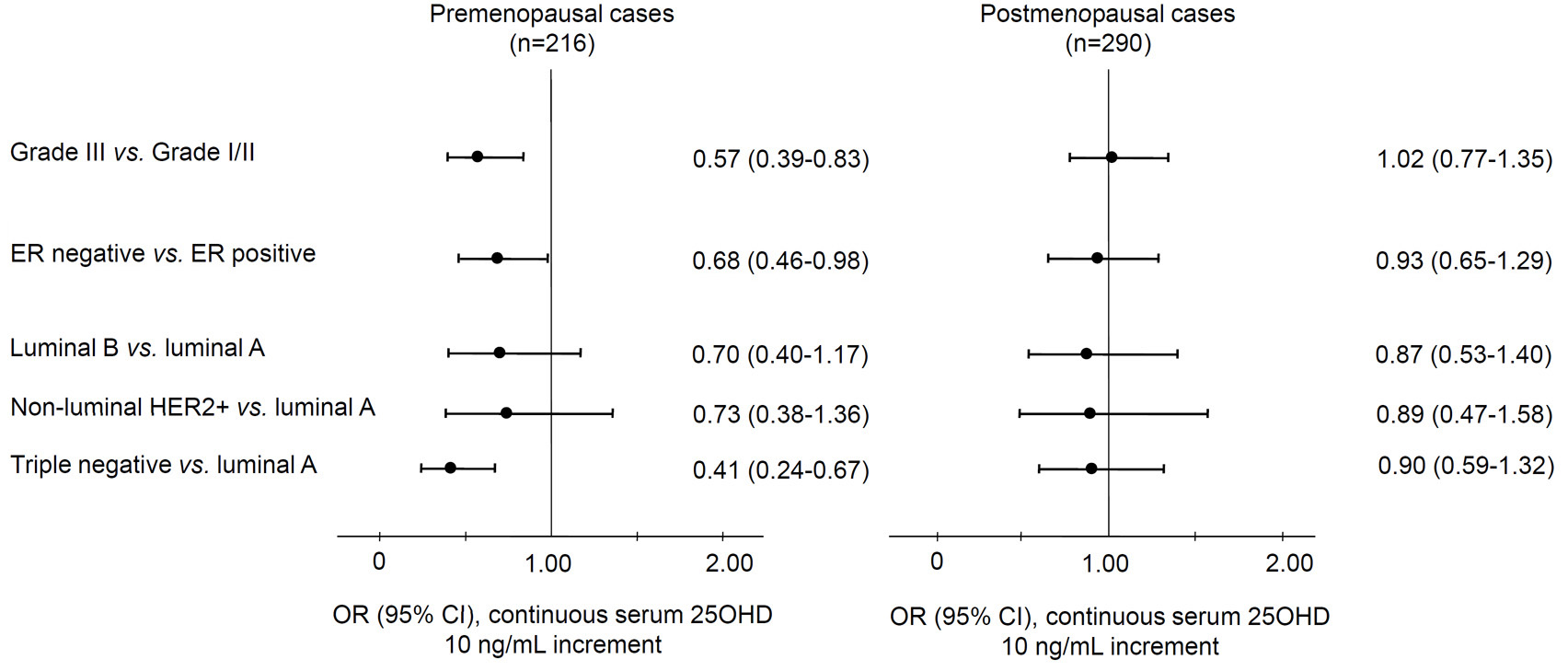

Supplement: Figure S2 — Case-only analysis of breast cancer risk by 10 ng/ml increase of vitamin D levels. Season-standardized serum 25-hydroxyvitamin D (25OHD) concentrations were entered into the regression models as a continuous variable. Odds ratios (OR) and 95% confidence intervals (CI) of an incremental increase of 10 ng/mL 25OHD were derived from multinomial logistic regression with adjustment for age at diagnosis and BMI, and presented in groups of tumor characteristics, where women with better prognostic characteristics (grade I/II, ER+, or luminal A subtype) were used as a referent group and women with carcinoma in situ (CIS) were excluded. Further adjustment for physical activity did not significantly change the results (data not shown). The lengths of horizontal lines are indicative of confidence intervals and the dots are indicative of odds ratios, with the corresponding odds ratios and 95% confidence interval given in numbers on the right of the Y-axis. (TIF) [file pone.0017251.s002.tif]
